# High prevalence of hypertension and cardiovascular disease risk factors among medical students at Makerere University College of Health Sciences, Kampala, Uganda

**DOI:** 10.1186/s13104-016-1924-7

**Published:** 2016-02-17

**Authors:** Kenneth V. Nyombi, Samuel Kizito, David Mukunya, Angella Nabukalu, Martin Bukama, Joseph Lunyera, Martha Asiimwe, Ivan Kimuli, Robert Kalyesubula

**Affiliations:** Clinical Epidemiology Unit, School of Medicine, Makerere University College of Health Sciences, Kampala, Uganda

## Abstract

**Background:**

Cardiovascular disease has become a leading global health challenge representing the largest cause of mortality in adults worldwide. Non communicable diseases are neglected in Uganda over infectious diseases. With increased urbanization, there is likely increase in burden of these NCDs yet there is paucity of reliable data regarding the NCD burden. We assessed the prevalence of hypertension and other cardiovascular disease risk factors among medical students at Makerere University, College of Health Sciences in Kampala, Uganda.

**Methods:**

We conducted a cross-sectional study at Makerere University comprising 180 medical students. We used a standardized questionnaire and anthropometric measurements to assess their cardiovascular disease risk factors using JNC-7. Logistic regression was used to assess factors associated with elevated blood pressure.

**Results:**

Of the 180 students surveyed, 107 (59 %) were males, mean age was 22 years (SD = 3 years), and 159 (88 %) were in their preclinical years of training. Cardiovascular risk factors with the highest prevalence were alcohol consumption (31.7 %); elevated systolic blood pressure (14 %); and excessive salt intake (13 %). Participants with elevated systolic blood pressure were more likely to be older (OR = 1.18), overweight (OR = 1.08), and with a personal history of cardiovascular disease (OR = 4.68).

**Conclusions:**

The prevalence of hypertension and known cardiovascular disease risk factors is high among the medical students. Strategies to prevent cardiovascular disease among the young population should be put in place.

## Background

Cardiovascular diseases (CVD) are among the leading causes of morbidity and mortality globally with hypertension ranking highest. About 80 % of the deaths from CVD occur among the low and middle-income earners in sub-Saharan Africa (SSA) [1, 2]. SSA is experiencing an epidemiological transition with a rapid increase in non-communicable diseases(NCDs) including CVD [3–6] which adds strain to the already complex health situation resulting from the infectious disease burden in the region [3, 7]. Unfortunately, attention to CVD risk factors as a form of prevention has been largely lacking in the region [8]. In Uganda, the prevalence of cardiovascular disease risk factors is high in both urban and rural populations [9–11]. However, few studies have been conducted to assess these factors among the young population.

Medical students have a role as future physicians and public health advocates in the management of cardiovascular disease and their cardiovascular disease related habits may predict their preparedness for this inevitable role [12, 13]. Studies determining the prevalence of cardiovascular risk factors among medical students elsewhere [13–18] have all shown high prevalence of cardiovascular risk factors.

In Uganda, national health programs currently prioritizing NCDs are still on a low scale. Most effort and funds are directed to infectious diseases such as HIV-AIDS, malaria and tuberculosis. The prevalence of non-communicable diseases and their associated risk factors have not been thoroughly studied especially among the young age groups. There is therefore scanty information regarding the prevalence of CVD among young population in Uganda. In this study, the aim was to determine the prevalence of cardiovascular disease risk and factors associated with high blood pressure among medical students at Makerere University, College of Health Sciences in Kampala, Uganda.

## Methods

### Study design

This was a cross-sectional study.

### Study setting

This study was conducted in April, 2013 at Makerere University College of Health Sciences (MakCHS), Kampala, Uganda. MakCHS is the oldest medical school in East Africa with an average number of 900 students. About one third of the students are in clinical years while the other two thirds are in pre-clinical years of training. The institution admits students from all the regions of the country, from both urban and rural high schools. The majority of the students (about 80 %) are accommodated in the five halls of residence at the university campus with the rest staying in the surrounding hostels. The University is surrounded by many suburbs with fast food restaurants. Students therefore often time opt for these fast foods in place of meals prepared in the halls of residence.

### Study participants and sample size

The sample size for the prevalence objective was calculated post hoc using Kish Leslie method of 1965 [19]. Where n = Z^2^pq/d ^2^ with Z = 1.96, p is the hypothesized population with hypertension which was 14.6 % [1] and d is a measure of the power of the study, probability of committing type 2 error, set at 0.05. It came to 191 participants while for the associated factors we used method of sample size calculation for two groups; we aimed for a power of 95 %, 0.05 chance of type 2 error. All medical students aged 18 years and above were invited to participate in the study by way of notices on school notice boards and brochures distributed in lecture rooms. Open invitations were extended to students at the data collection sites on the medical school campus. Out of the students who responded to your advertisements to participate, none declined to participate upon being given full details of the study. All medical students who were eligible and consented to the study were included in our sample using consecutive sampling until no more participants could be enrolled.

### Variables and data collection

A standardized semi-structured questionnaire was used to capture demographic information and named cardiovascular disease risk factors including: history of smoking cigarette; alcohol consumption; involvement in aerobic exercises; personal and family history of cardiovascular diseases. The weight of the participants was measured using a calibrated Secca weighing scale to the nearest 0.5 kg with the participant not wearing shoes. The height was taken using a stadiometer to the nearest 0.5 cm with the participant standing upright with the heel, buttock and upper back along the same vertical plane. Body mass index (BMI) was calculated by dividing the weight in kilograms by the square of the height in meters.

Overweight and obesity were defined as a BMI between 25 and 30 and BMI of 30 and above, respectively. Blood pressure was measured using a calibrated sphygmomanometer. Three consecutive readings of blood pressure were taken following a 5 min rest to allow the participants vitals return to their at-rest values. The blood pressure was read to the nearest millimeter of mercury with the participant seated, and a mean reading was calculated from the 3 readings. Hypertension was defined as systolic BP of 140 mmHg or more and/or diastolic of 90 mmHg or more, according to the7th Joint National Committee of High BP (JNC-7) [20]. Pre hypertension was defined as a systolic BP of 130–139 and/or a diastolic BP of 85–89, according to the JNC-7 [20].

The waist circumference of the participant was taken using a non-stretch tape measure placed at a point midway between the lowest rib and the iliac crest on bear skin. Hip circumference measured at the level of the greater trochanter using a non-stretch tape measure. The waist-hip ratio (WHR) was then calculated. Excessive body fat was defined as a waist-hip ratio above 0.90 in males and 0.85 in females. Random blood sugar was determined using a glucometer before the participants had their lunch. Diabetes mellitus and impaired glucose tolerance were defined by a random blood sugar of >200 and >126 mg/dl respectively. Measurements were taken by the principal investigator and well trained co-investigators with the help of a trained laboratory technician. Both diabetes mellitus and impaired glucose intolerance were defined as dysglycemia in this study.

### Data management and analysis

Data was double entered using Epidata 3.1 (www.epidata.dk) and analyzed using STATA version 12. Continuous data was summarized using means and standard deviations, medians and interquartile ranges depending on the distribution of the data. Categorical data was summarized using percentages and proportions. Prevalence of hypertension was calculated as a proportion. Comparison was made between participants who had hypertension and those without hypertension. Comparisons for continuous variables were made using the Student *t* test or the Mann–Whitney U (Wilcoxon rank sum test) depending on the distribution, while categorical data were compared using the Chi square or Fisher’s exact test. Logistic regression was used to establish the association between the independent variables and blood pressure. All variables that had P value of ≤0.2 at bivariate analysis were included in the stepwise multivariate analysis together with those variables that are known risk factors from the literature search performed. Interactions were assessed using Chunk test.

### Ethical consideration

The research was approved by the Makerere University College of Health Sciences School of Medicine Research and Ethics committee. Written informed consent was obtained from the study participants prior to enrollment into the study. Individual cardiovascular risk factor results were given to the respective individuals and appropriate referrals made.

## Results

A total of 180 medical students of the intended 191 (94.2 %) participated in the study. This is 20 % of the total population of medical students (n = 900). All of the participants completed the study procedures. Of these, 107 (59.4 %) were males, the mean age was 22 ± 3 years, 159 (88.3 %) of the subjects were from pre-clinical years. The demographic characteristics are shown in Table 1.

**Table 1:** Socio-demographic characteristics of 180 medical students at Makerere CHS, 2013

Variables	Categories	Value
Mean age (years) ± SD		21.6 ± 3.3
Body mass index	Underweight	9 (5.0)
	Normal	155 (86.1)
	Overweight	14 (7.8)
	Obese	2 (1.1)
Mean height (m) ± SD		1.73 ± 1.04
Sex	Male	107 (59.4)
	Female	73 (41.6)
Systolic blood pressure (mmHg)	Below 130	154 (85.6)
	130–139	16 (8.9)
	140 and above	10 (5.6)
Diastolic blood pressure (mmHg)	Below 85	119 (66.1)
	85 to 89	25 (13.9)
	90 and above	36 (20.0)
Year of study	Year one	59 (32.8)
	Year two	74 (41.1)
	Year three	26 (14.4)
	Year four	18 (10.0)
	Year five	3 (1.7)
Mean waist-hip ratio ± SD	Normal	167 (92.8)
	Excessive body fat	13 (7.2)
Mean random blood sugar (mmol/l)	Normal	171 (95.0)
	Altered glucose tolerance	6 (3.3)
	Diabetic	3 (1.7)

Except where otherwise stated, the values are numbers (percentages) of participants

The number of participants with elevated blood pressure was 59 giving a prevalence of 32.8 %.

### Cardiovascular disease risk factor

Mean weight of the participants was 59.9 ± 8.6 kg and 86.1 % (155/180) had normal BMI; 92.8 % (167/180) had normal waist-hip ratio as shown in Table 1.

Overall 13 (12.2 %) and 22 (7.2 %) of the 180 participants had a family history of hypertension only and diabetes only respectively while 8 (4.4 %) had family history of both illnesses. The majority, 179 (97.8 %) of the 180 participants investigated had no history of smoking. Close to one third of the 180 participants, 57 (31.7 %) reported drinking alcohol and of these, majorities (72.4 %) were male. Of the 180 participants, 6 (3.3 %) had pre-diabetic blood sugar levels while 3 (1.7 %) were in diabetic range (Table 1). Additionally, 162 (90 %) had normal BMI (171) 95.00 % had normal random blood sugar as shown in Table 2.

**Table 2:** Cardiovascular disease risk factors among 180 medical students at MakCHS 2013

Variables	Categories	Number (column percentages)		
Year of study		Pre-clinical (159)	Clinical (21)	Overall (180)
No smoking		155 (97.5)	21 (100.0)	176 (97.8)
Taking alcohol		43 (27.0)	14 (66.7)	57 (31.7)
Salt intake	Little	28 (17.6)	3 (14.3)	31 (17.2)
	Moderate	109 (68.6)	15 (71.4)	126 (70.0)
	A lot	22 (13.8)	0 (00.0)	23 (12.8)
Body mass index	Normal	143 (89.9)	19 (90.5)	162 (90.0)
	Abnormal	16 (10.1)	2 (9.5)	18 (10.0)
Random blood sugar	Normal	151 (95.0)	21 (100.0)	171 (95.0)
	Elevated	8 (5.0)	0 (0.0)	9 (5.0)
Family history	Diabetes mellitus	9 (5.7)	4 (19.1)	13 (7.2)
	Hypertension	18 (11.3)	4 (19.1)	22 (12.2)
	Diabetes and hypertension	8 (5.0)	0 (0.0)	8 (4.4)
	Others	12 (7.5)	2 (9.5)	14 (7.8)
	None	112 (70.4)	11 (52.4)	123 (68.3)
No personal history^a^		154 (96.9)	19 (90.5)	4 (2.2)

^a^Personal history of cardiovascular disease

### Overall Blood pressure and the factors associated

The mean systolic and diastolic blood pressures for the respondents were 114 ± 12 and 78 ± 10 respectively. Of the 180 participants, 25 (14 %) had hypertensive ranges of blood pressure. Participants with elevated systolic blood pressure were more likely to be older (OR = 1.18), overweight (OR = 1.082), and with a personal of cardiovascular disease (OR = 4.68.) as shown in Table 3. Significant differences between participants with elevated blood pressure and their normal counterparts were in weight, height and family history of hypertension as shown in Table 4.

**Table 3:** Table for the bivariate and multivariate analysis the factors that predict blood pressure among 180 MakCHS students, 2013

Variables	Bivariate analysis			Multivariate analysis		
	OR	95 % CI	P value	OR	95 % CI	P value
Age	1.176	1.057–1.308	0.003^*^	1.178	1.030–1.346	0.016^*^
Year of study	1.001	0.906–1.106	0.987			
No CVD history	1	–	–	1	–	–
CVD history	4.069	1.114–14.869	0.034^*^	4.676	1.244–17.582	0.022^*^
Family history of CVD	1	–	–	1	–	–
No Family history of CVD	0.963	0.735–1.262	0.787^**^	0.833	0.597–1.164	0.285
Non smoker	1	–	–			
Smoking	2.088	0.287–15.200	0.467^**^			
Non alcoholic	1	–	–	1	–	–
Alcohol intake	1.164	0.600–2.260	0.650^**^	1.058	0.504–2.218	0.882
Frequency of alcohol intake	2.000	0.438–9.128	0.371^**^			
Salt intake	0.688	0.386–1.227	0.205^**^			
Weight	1.097	1.050–1.147	0.000^*^	1.082	1.033–1.134	0.001^*^
Height	0.917	0.602–1.394	0.684			
Body mass index	1.108	0.990–1.240	0.075^*^			
Waist circumference	1.028	0.988–1.070	0.179^*^			
Hip circumference	0.986	0.951–1.022	0.443			
Random blood sugar	0.984	0.919–1.054	0.647			

^*^ Variable showing significance of P < 0.2

^**^ Variables that will be included in multivariate analysis although they have P value >0.2 since they are known risk factors for CVD from literature

**Table 4:** Comparing factors that predict blood pressure among 180 students at MakCHS, 2013

Variables	Categories	Normal BP (121)	High BP (59)	
		Value	Value	P value
ValueAge		20.99 ± 2.73	22.75 ± 4.05	0.001^a^
Mean weight		58.00 ± 8.40	64.00 ± 7.61	0.000^a^
Body mass index		21.59 ± 2.93	22.41 ± 2.51	0.668
Waist circumference		70.31 ± 9.26	72.42 ± 10.59	0.174
Hip circumference		94.01 ± 11.91	92.64 ± 8.00	0.427
Waist-hip ratio		0.76 ± 0.06	0.79 ± 0.06	0.001^a^
Random blood sugar		5.59 ± 4.17	5.08 ± 2.42	0.627
Year of study	Year one	4 (5.00)	18 (31.03)	0.694
	Year two	49 (61.25)	22 (37.93)	
	Year three	15 (18.75)	11 (18.97)	
	Year four	11 (13.75)	6 (10.34)	
	Year five	1 (1.25)	1 (1.72)	
Personal history	None	119 (99.17)	53 (89.83)	0.006
	Hypertension	0 (00.00)	3 (5.08)	
	Others	1 (0.83)	3 (5.08)	
Family history	None	82 (67.77)	41 (69.49)	0.988
	Hypertension	15 (12.40)	7 (11.86)	
	Diabetes mellitus	9 (7.44)	4 (6.78)	
	Both HTN and DM^a^	9 (7.44)	5 (8.47)	
	Others	6 (4.97)	2 (3.39)	
History of treatment	None	118 (97.52)	55 (93.22)	0.042
	Hypertension	0 (0.00)	3 (5.08)	
	Others	3 (2.48)	1 (1.69)	
No smoking		119 (98.35)	57 (96.61)	0.598
Alcohol intake		37 (30.58)	20 (33.09)	0.653
Salt intake	Little	18 (14.88)	13 (22.03)	0.426
	Moderate	86 (71.07)	40 (67.80)	
	A lot	17 (14.05)	6 (10.17)	

*HTN* hypertension

^a^ *DM* diabetes mellitus

## Discussion

Our study showed a high prevalence of both pre-hypertension and hypertension of 18.8 and 14 % respectively. The high prevalence could be as a result of change in lifestyle and increased urbanization. This causes increased sedentary lifestyles and intake of fortified foods [21]. This prevalence is comparable to results from other studies in the region, all showing high prevalence of cardiovascular disease risk factors [1, 2, 4, 22–25]. However unlike in the studies above which were conducted generally among adults, the subjects in our study were young people (mean age 22 years SD ± 3 years). This raises a concern that it hypertension and associated CVD risk factors are creeping into the young age bracket and over time putting the future generation at an increased risk of CVDs that may increase morbidity and decrease productivity. Further still, our subjects were medical students who are assumed to be knowledgeable about cardiovascular disease prevention. This therefore suggests that the prevalence of NCD in the developing world may be increasing and is not sparing the young age groups. The factors associated with hypertension were studied and they included; increased weight, older age and personal or family history of cardiovascular disease. These same associations were demonstrated in several other studies [1, 2, 4, 21, 23, 24, 26–29]. It is known that that increasing age causes numerous changes in the cardiovascular system like increased vessel stiffness and increased body mass [22, 30]. Increased weight is associated with cardiovascular system changes [28] like fat deposition in the vessel lumen which eventually lead to elevated blood pressures. However, we could have underestimated the prevalence of hypertension and pre-hypertension in our study because the proportion of students in clinical years (older students and therefore more likely to be hypertensive) sampled was low.

Alcohol consumption was the most prevalent modifiable risk factor for CVD among the respondents at 31.7 %. Similar figures have been reported elsewhere [26]. It would have been expected that since medical students have knowledge about the dangers alcohol consumption carries with respect to CVD, their consumption rate would be lower. This high alcohol consumption rate among medical students poses questions about the preparedness of these future physicians in curbing NCDs. However, alcohol consumption could have been overestimated in our study because we did not quantify the amounts taken.

In our study, the prevalence of overweight and obesity combined was 9.4 %. Although the figures are in rhyme with similar studies in other developing countries [21], elevated BMI was not however associated with hypertension as observed in other studies [21, 26].

In our study, the prevalence of dysglycemia (diabetic and pre-diabetic sugar levels) was 5 %. These were young healthy persons without features of type I diabetes. This means these young persons are at risk of type II diabetes. The prevalence would probably have been slightly higher if older students had been surveyed. This therefore means that clinicians should actively screen young persons for diabetes and other metabolic diseases.

Our study had some limitations: for some variables we relied on self-report like the history of cigarette smoking and alcohol consumption which is prone to bias, we used a small sample size which could have affected the precision of the measurements, no fasting blood sugar was done to assess for diabetes but only the random blood sugar. Basing on the nature of recruiting participants into the study, it could have created a selection bias since participants who are healthy are more likely to participate in the study. The intended sample size was not achieved. This could have affected the accuracy in our estimates. Medical students are a population that is relatively young, elite and knowledgeable about hypertension therefore findings from our work may have limited generalizability to the general population that is not elite and relatively older.

## Conclusions

This study conducted among medical students has shown a high prevalence of pre hypertension and hypertension among this young population of participants. The prevalence of dysglycemia was also high among this young population of participants. This means that the burden of NCDs is high among the young population contrary to what was documented before. The results from our study reveal that the young people are at high risk for NCD. It is therefore imperative that clinicians start to actively screen for these diseases during their routine interactions with this population.

Additionally, public health endeavors on prevention of NCDs be brought on board in addition to those targeting the communicable disease.

Finally, more studies including national surveys should be conducted especially among the young age groups, to unmask the indolent burden of hypertension in Uganda.

## Abbreviations

BMI: body mass index
BP: blood pressure
CVD: cardiovascular disease
MakCHS: Makerere University College of Health Sciences
NCDs: non-communicable Diseases
RBS: random blood sugar
WHR: waist-hip ratio
